# Tyrosine kinase inhibitors relax pulmonary arteries in human and murine precision-cut lung slices

**DOI:** 10.1186/s12931-019-1074-2

**Published:** 2019-06-06

**Authors:** Annette D. Rieg, Nina A. Bünting, Christian Cranen, Said Suleiman, Jan W. Spillner, Heike Schnöring, Thomas Schröder, Saskia von Stillfried, Till Braunschweig, Paul W. Manley, Gereon Schälte, Rolf Rossaint, Stefan Uhlig, Christian Martin

**Affiliations:** 1 Department of Anaesthesiology, Medical Faculty Aachen, RWTH-Aachen, Aachen, Germany; 2 Institute of Pharmacology and Toxicology, Medical Faculty Aachen, RWTH-Aachen, Aachen, Germany; 3 Department of Cardiac and Thoracic Surgery, Medical Faculty Aachen, RWTH-Aachen, Aachen, Germany; 4 Department of Surgery, Luisenhospital Aachen, Aachen, Germany; 5 Institute of Pathology, Medical Faculty Aachen, RWTH-Aachen, Aachen, Germany; 6 Novartis Pharma AG, Basel, Switzerland

**Keywords:** Tyrosine kinase inhibitors, Imatinib, Nilotinib, Pulmonary arteries, Pulmonary arterial hypertension

## Abstract

**Background:**

Tyrosine kinase inhibitors (TKIs) inhibit the platelet derived growth factor receptor (PDGFR) and gain increasing significance in the therapy of proliferative diseases, e.g. pulmonary arterial hypertension (PAH). Moreover, TKIs relax pulmonary vessels of rats and guinea pigs. So far, it is unknown, whether TKIs exert relaxation in human and murine pulmonary vessels. Thus, we studied the effects of TKIs and the PDGFR-agonist PDGF-BB in precision-cut lung slices (PCLS) from both species.

**Methods:**

The vascular effects of imatinib (mice/human) or nilotinib (human) were studied in Endothelin-1 (ET-1) pre-constricted pulmonary arteries (PAs) or veins (PVs) by videomicroscopy. Baseline initial vessel area (IVA) was defined as 100%. With regard to TKI-induced relaxation, K^+^-channel activation was studied in human PAs (PCLS) and imatinib/nilotinib-related changes of cAMP and cGMP were analysed in human PAs/PVs (ELISA). Finally, the contractile potency of PDGF-BB was explored in PCLS (mice/human).

**Results:**

Murine PCLS: Imatinib (10 μM) relaxed ET-1-pre-constricted PAs to 167% of IVA. Vice versa, 100 nM PDGF-BB contracted PAs to 60% of IVA and pre-treatment with imatinib or amlodipine prevented PDGF-BB-induced contraction. Murine PVs reacted only slightly to imatinib or PDGF-BB. Human PCLS: 100 μM imatinib or nilotinib relaxed ET-1-pre-constricted PAs to 166% or 145% of IVA, respectively, due to the activation of K_ATP_-, BK_Ca_^2+^- or K_v_-channels. In PVs, imatinib exerted only slight relaxation and nilotinib had no effect. Imatinib and nilotinib increased cAMP in human PAs, but not in PVs. In addition, PDGF-BB contracted human PAs/PVs, which was prevented by imatinib.

**Conclusions:**

TKIs relax pre-constricted PAs/PVs from both, mice and humans. In human PAs, the activation of K^+^-channels and the generation of cAMP are relevant for TKI-induced relaxation. Vice versa, PDGF-BB contracts PAs/PVs (human/mice) due to PDGFR. In murine PAs, PDGF-BB-induced contraction depends on intracellular calcium. So, PDGFR regulates the tone of PAs/PVs. Since TKIs combine relaxant and antiproliferative effects, they may be promising in therapy of PAH.

## Background

Pulmonary arterial hypertension (PAH) is characterised by increased pulmonary vascular tone and remodelling of all vessel layers, e.g. intima, media and adventitia of the pulmonary vascular bed [1, 2]. So far, PAH goes along with high mortality strongly depending on the underlying risk factors and the WHO functional class [3]. According to this, the arrest of disease progress appears to be essential to extend life time. With this regard, antiproliferative agents are of high clinical impact in PAH [4]. Recently, tyrosine kinase inhibitors (TKIs) have been proven to attenuate or prevent the pulmonary vascular remodelling by its inhibitory action on the platelet-derived growth factor receptor (PDGFR) [5–14]. Beyond that, a few studies in rats [15, 16] and guinea pigs [17] have shown that the TKIs imatinib [15–17], sorafenib [15] and nilotinib [15] exert considerable relaxation in pulmonary arteries (PAs) [15, 16] and veins (PVs) [17]. PDGFR-inhibition, as a new therapeutic approach in PAH appears to be even more convincing, as the PDGFR-agonist PDGF-BB mediates aside proliferation also contraction, assigning PDGFR a central role in disease progress [5, 14, 18–20].

Thusfar, it is unclear whether TKI- or imatinib-induced relaxation represents a basic and widespread phenome, operable across all species, e.g. in mice or humans. Whereas the IMPRES study revealed remarkable imatinib-related pulmonary haemodynamic benefits in advanced PAH [10], considerable side effects such as pleural effusions, QTc prolongation or subdural haematoma also were reported [10, 21]. Apart from that, some TKIs primarily dasatinib [22–25], but also bosutinib [23, 25], sorafenib [26] or ponatinib [25, 27] exert toxic effects on the pulmonary vascular bed and even worsen PAH. Therefore, it would be beneficial to identify alternative TKIs which target both, the pulmonary vascular tone and the remodelling without exerting pulmonary vascular toxicity [25, 26]. Nilotinib might represent such an alternative TKI, as it has been shown to act antiproliferative in smooth muscle cells (SMCs) from human PAs [28] and to relax rat PAs [15]. Until now it has been unclear, whether nilotinib also relaxes the human pulmonary vascular bed.

To investigate these topics, we studied the relaxant effect of imatinib in precision-cut lung slices (PCLS) from mice and men and also evaluated the relaxant potential of nilotinib in human PCLS. We analysed, whether K^+^-channel activation contributes to the relaxant effect of imatinib/nilotinib, as it was shown for imatinib in PVs from guinea pigs [17]. Beyond that, we studied the influence of imatinib/nilotinib on intracellular cAMP/cGMP in human PAs/PVs. Last, we analysed the contractile effects of PDGF-BB in pulmonary vessels (mice/men) and evaluated, whether this contraction is preventable by imatinib [17, 20].

The investigation was performed by the use of PCLS, a well-established method [17, 29–32] that allows PAs, PVs and airways to be investigated within their natural tissue anatomy [33]. PCLS are designated by a further strength; they enable to perform interspecies comparison [30–32]. This aspect is of particular value, as human lung tissue is quite limited and thus, it often only serves as a “proof of principle”. However, due to the known interspecies differences [32] and a possible relevance of TKI-induced relaxation for the management of PAH [13], we primarily performed our study in human PCLS.

## Methods

### Animals and patients

Female BALB/c mice (20 ± 3 g) were obtained from Charles River (Sulzfeld, Germany). All animal studies were approved by the Landesamt für Natur, Umwelt und Verbraucherschutz Nordrhein-Westfalen (ID: 8.87–51.05.20.10.245 and 84–02.04.2013A146) and performed after the Directive 2010/63/EU of the European Parliament. Human lung tissue was obtained from patients undergoing lobectomy due to cancer. The study was performed according to the Declaration of Helsinki and approved by the local ethics committee (EK 61/09). All patients gave written informed consent. After macroscopic inspection and palpation by a pathologist, tumor-free human tissue was obtained. None of the patients showed any sign of PAH (echocardiography, histology).

### PCLS

Murine PCLS were prepared as described [29] including some modifications. After terminal anaesthesia and exsanguination, the trachea was cannulated, the diaphragm and the thorax cavity were opened, the PA was cannulated and the left ventricle was disclosed by an incision. Porcine skin gelatin (6%) was instilled via the pulmonary arterial cannula to wash out the blood and to stabilise the pulmonary vascular bed. Then, the ventricular incision and PA cannula were closed to prevent leakage of the gelatin from the pulmonary vascular bed. Next, murine lungs were filled via the trachea with 1.5% low melting agarose. Human lungs [31] were filled via a main bronchus with 1.5% low melting agarose. To harden murine and human agarose-filled lungs, they were cooled with ice. Then, tissue cores (diameter 11 mm) were prepared and cut into about 300 μM thick slices with a Krumdieck tissue slicer (Alabama Research & Development, Munford, AL, USA). PCLS were incubated at 37 °C and repeated medium changes were performed to wash out the agarose.

### Identification of the vessels, histology

**Fig. 1:**
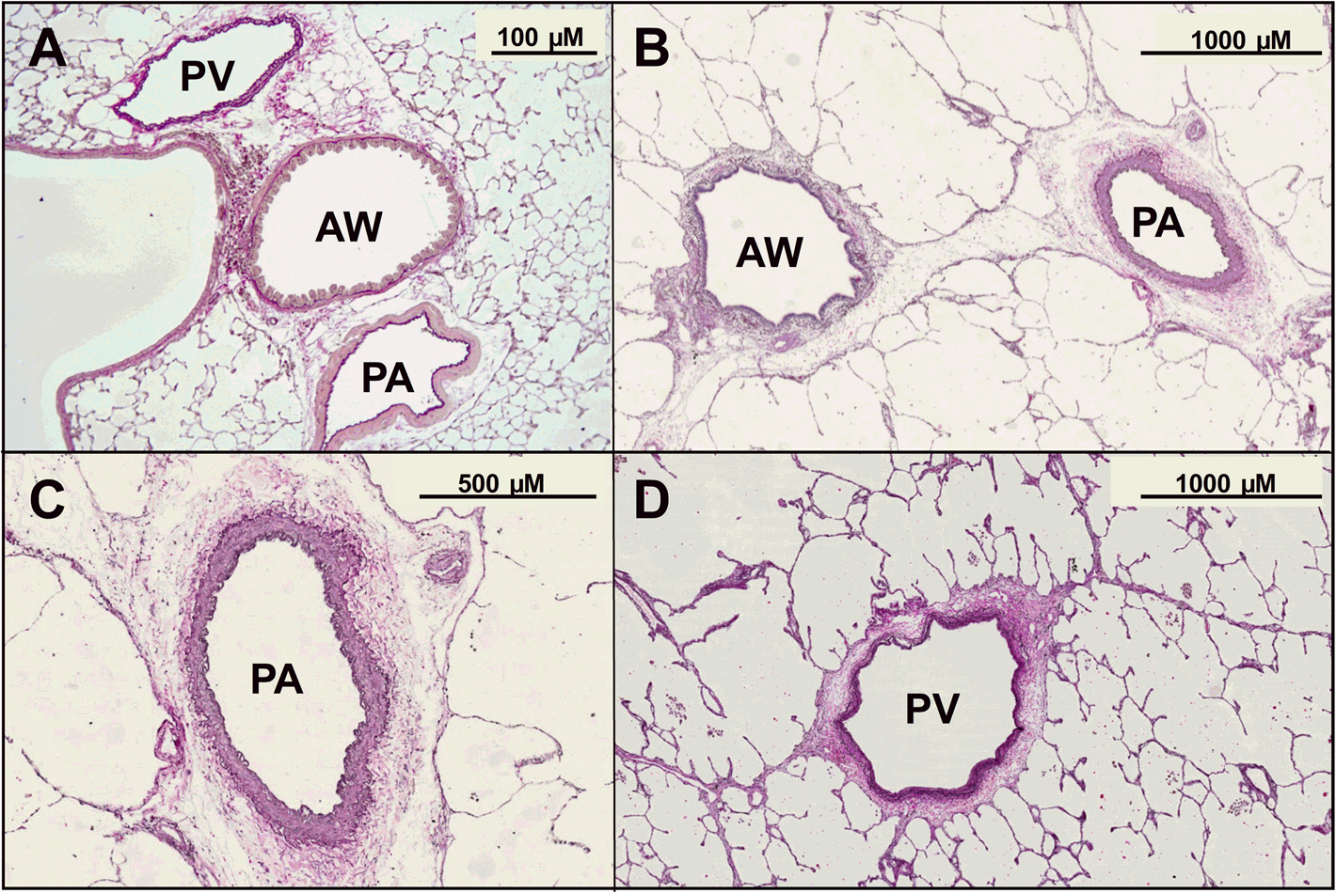
Histology of murine and human PCLS. **a** Murine PCLS: The PA is located aside the airway (AW) and characterised by a thick media, whereas the PV lies more aside. **b** Human PCLS**:** The PA is located near the AW. **c** Human PCLS**:** PA with typical elastica interna and externa. **d** Human PCLS**:** PV with elastica interna

PAs and PVs were discriminated by their anatomical landmarks; PAs are located adjacent to airways, while PVs lie aside. After termination of the experiments, PCLS from both species were stained with Elastica van Gieson (EVG) staining to confirm their arterial or venous assignment [31] (Fig. 1).

### Pre-constriction, pharmacological interventions, measurements, and videomicroscopy

Vessels often only relax, if they are pre-constricted. Therefore, we pre-constricted murine and human PAs and PVs with Endothelin-1 (ET-1). Prior to the measurement, murine airways were cut by a scalpel, because intact airways strongly contract in response to ET-1 and possibly influence thereby the vessel tone of murine PAs/PVs indirectly due to their close proximity.

**Fig. 2:**
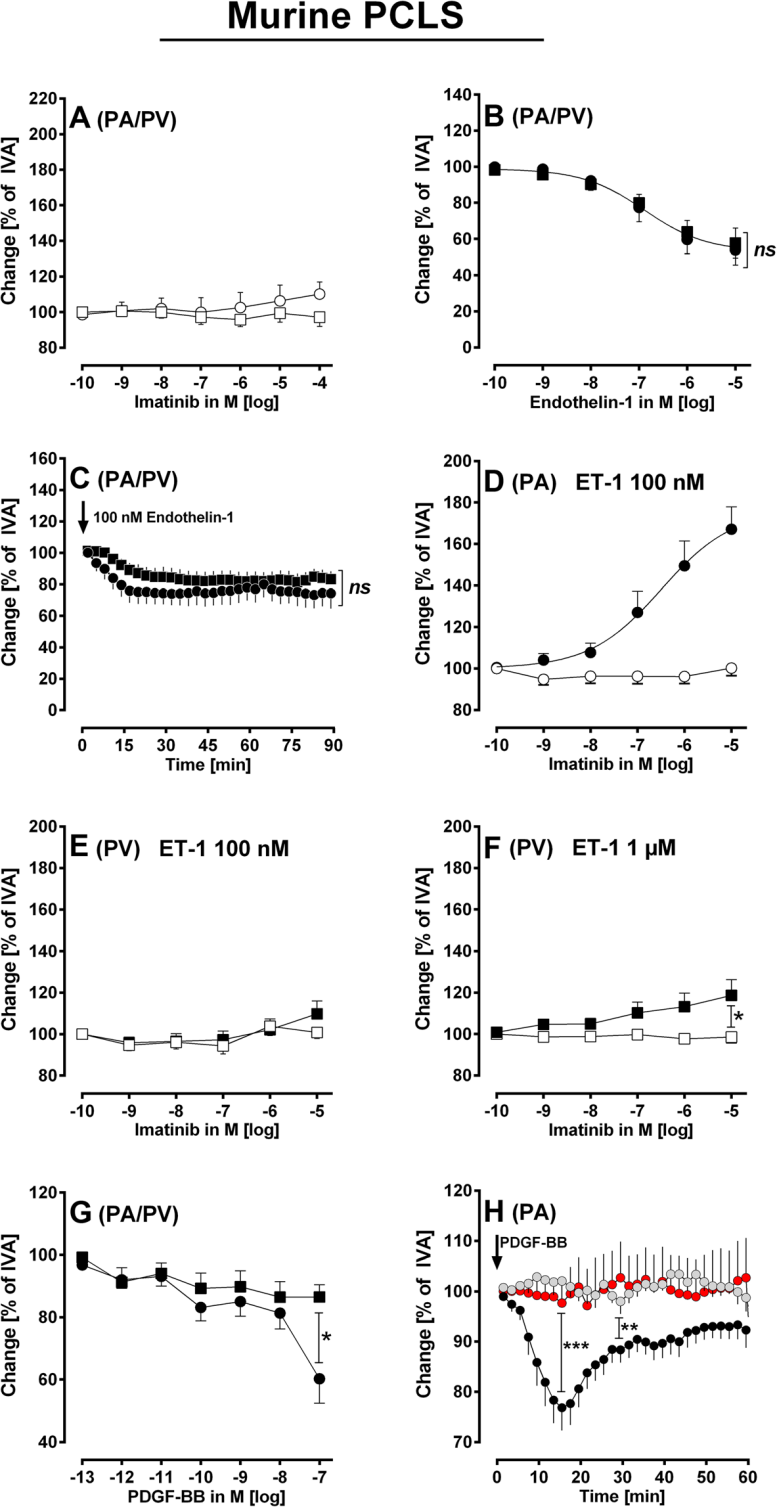
Murine PCLS. **a** Concentration-response curve of imatinib in naïve PAs and PVs: (◯) PAs (*n* = 6); (□) PVs (*n* = 7). **b** Concentration-response curve of ET-1 in naïve murine pulmonary vessels: (●) PA (*n* = 7); (■) PV (*n* = 6). **c** The contractile effect of 100 nM ET-1 in PAs and PVs: (●) PA (*n* = 4); (■) PV (*n* = 5). **d** Effect of increasing concentrations of imatinib in pre-constricted PAs: (◯) 100 nM ET-1 (*n* = 8); (●) 100 nM ET-1 / imatinib (*n* = 10). **e** Effect of increasing concentrations of imatinib in pre-constricted PVs: (□) 100 nM ET-1 (*n* = 8); (■) 100 nM ET-1 / imatinib (*n* = 10). **f** Effect of increasing concentrations of imatinib in pre-constricted PVs: (□) 1 μM ET-1 (*n* = 7); (■) 1 μM ET-1 / imatinib (*n* = 9). **g** Concentration-response of PDGF-BB in murine PAs and PVs: (●) PA (*n* = 10); (■) PV (*n* = 7). **h** The contractile effect of PDGF-BB in PAs: (●) 100 nM PDGF-BB (*n* = 12); () 1 μM imatinib / 100 nM PDGF-BB (*n* = 7); () 100 nM amlodipine / 100 nM PDGF-BB (n = 5). **b**, **d** EC_50_ values were calculated using the standard 4-paramter logistic non-linear regression model. **c**, **h** Statistics was performed by a linear mixed model analysis (LMM). **f**, **g** Statistics was performed by the Mann-Whitney U test. *P* < 0.05 are considered as significant: ^*^ *p* < 0.05,^**^ *p* < 0.01 and ^***^ *p* < 0.001

**Fig. 3:**
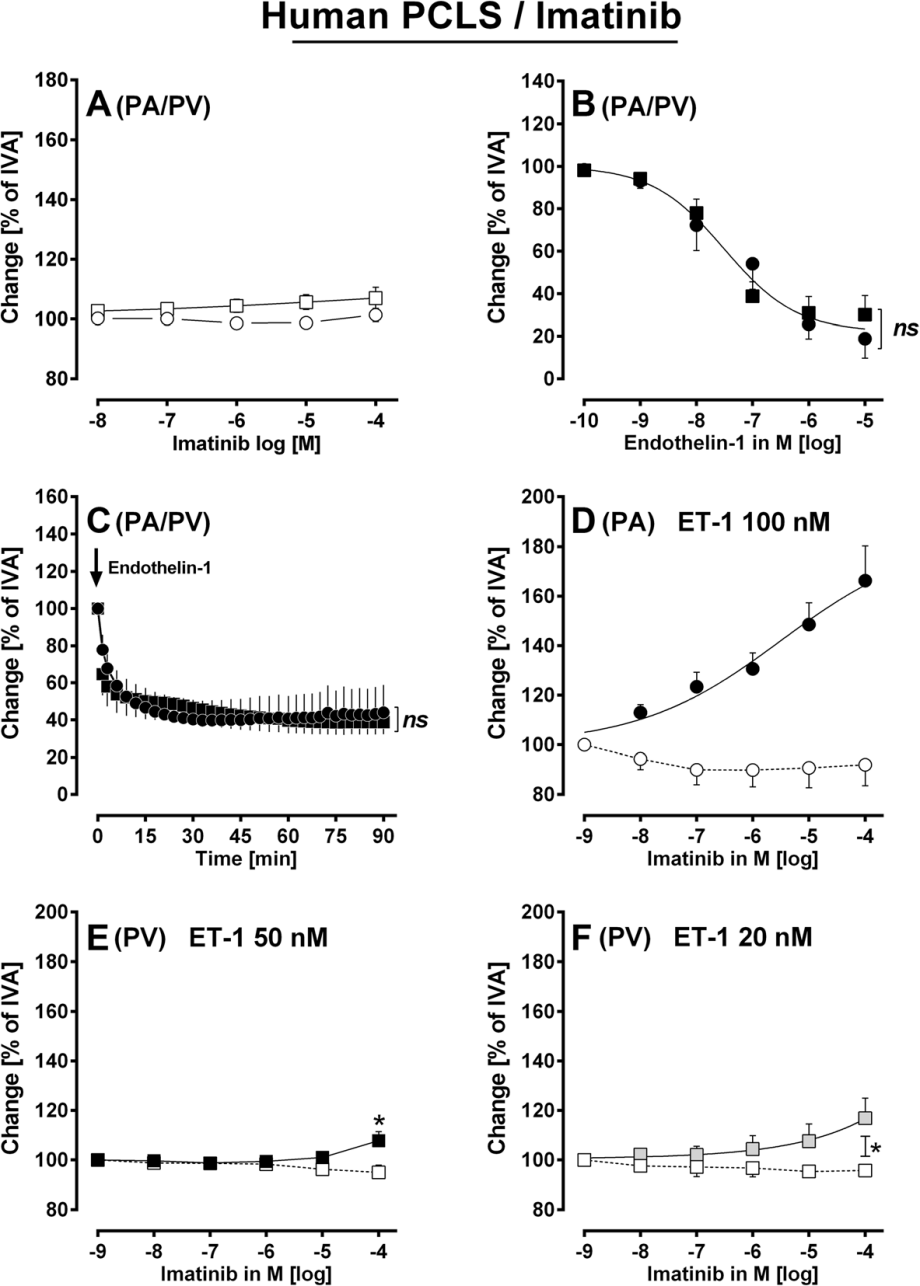
Vascular effects of Endothelin-1 and imatinib in human pulmonary vessels. **a** Imatinib in naïve PAs/PVs: (◯) PA (*n* = 4); (□) PV (*n* = 6). **b** Concentrations-response curve of ET-1 in naïve human pulmonary vessels: (●) PA (*n* = 7); (■) PV (*n* = 6). **c** ET-1 in human PAs/PVs: (●) PA 100 nM ET-1 (*n* = 4); (■) PV 50 nM ET-1 (*n* = 6). **d** Effect of increasing concentration of imatinib in pre-constricted PAs: (◯) 100 nM ET-1 (*n* = 7); (●) 100 nM ET-1 / imatinib (*n* = 10). **e** Effect of increasing concentration of imatinib in pre-constricted PVs: (□) 50 nM ET-1 (*n* = 6); (■) 50 nM ET-1 / imatinib (*n* = 6). **f** Effect of increasing concentration of imatinib in pre-constricted PVs: (□) 20 nM ET-1 (*n* = 6); () 20 nM ET-1 / imatinib (*n* = 4) **b**, **d** EC_50_ values were calculated using the standard 4-paramter logistic non-linear regression model. **c)** Statistics was performed by a LMM. **e**, **f** Statistics was performed by the Mann-Whitney U test. *P* < 0.05 are considered as significant: ^*^ *p* < 0.05, ^**^ *p* < 0.01 and ^***^ *p* < 0.001

**Fig. 4:**
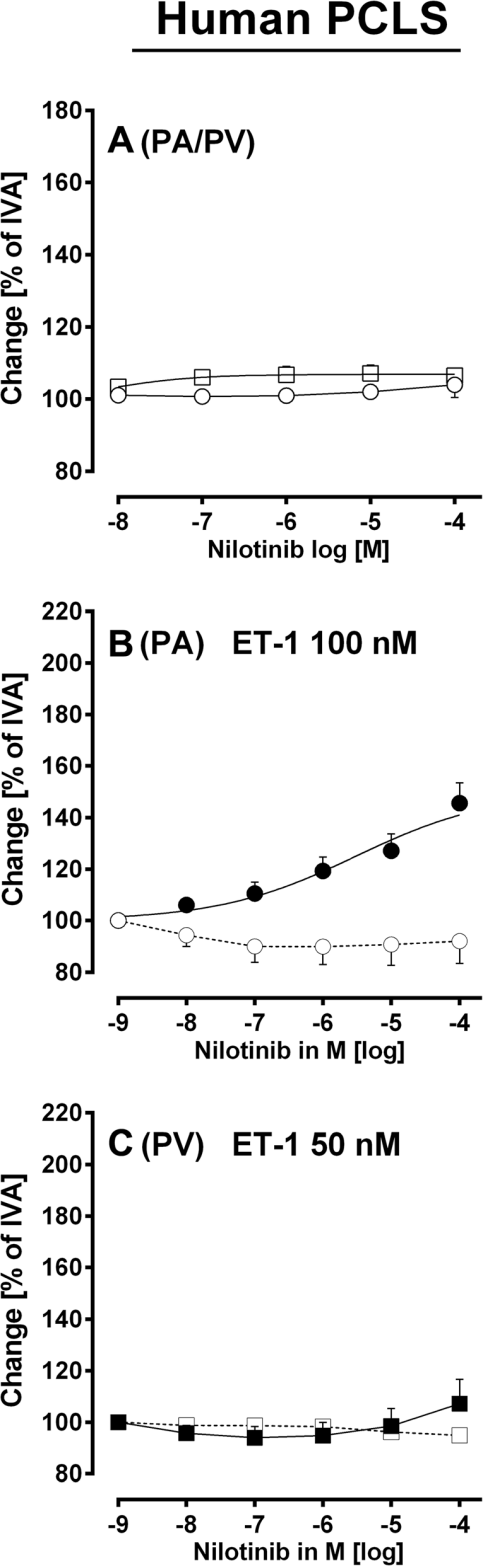
Vascular effects of nilotinib in human pulmonary vessels. **a** Nilotinib in naïve PAs/PVs (◯) PA (*n* = 5); (□) PV (*n* = 6). **b** Effect of increasing concentration of nilotinib in pre-constricted PAs: (◯) 100 nM ET-1 (*n* = 7); (●) 100 nM ET-1 / imatinib (*n* = 10). **c** Effect of increasing concentration of nilotinib in pre-constricted PVs: (□) 50 nM ET-1 (*n* = 6); (■) 50 nM ET-1 / imatinib (*n* = 8). **b** EC_50_ value was calculated using the standard 4-paramter logistic non-linear regression model. *P* < 0.05 are considered as significant: ^*^ *p* < 0.05, ^**^ *p* < 0.01 and ^***^ *p* < 0.001

To achieve comparable degrees of pre-constriction, murine PAs/PVs (Fig. 2b, c) and human PAs (Fig. 3b, c) were incubated with 100 nM ET-1, whereas humans PVs were incubated with 50 nM ET-1 (Fig. 3b, c). These concentrations of ET-1 produced stable contractions after 30 min (Figs. 2c and 3c). In addition, murine PVs were also pre-constricted with 1 μM ET-1 (Fig. 2f) and human PVs were also pre-constricted with 20 nM ET-1 (Fig. 3f) to challenge imatinib-induced relaxation. After pre-constriction, PAs and PVs from mice (Fig. 2d-f) and humans (Fig. 3d-f and Fig. 4b, c) were exposed to increasing concentrations of imatinib or nilotinib and concentration-response curves were performed.

In humans PAs, the involvement of K^+^-channel-activation within imatinib- or nilotinib-induced relaxation was evaluated. Therefore, PCLS were pre-constricted with ET-1 and additionally pre-treated with one of the following inhibitors: K_ATP_-channel (10 μM glibenclamide); BK_Ca_^2+^-channel (100 nM iberiotoxin); K_v_-channel (5 mM 4-aminopyridine (4-AP)); subsequently, concentration-response curves with imatinib and nilotinib were performed. Changes of the initial vessel area (IVA) were quantified in % and are reported as “Change [% of IVA]”. Hence, a vessel area < 100% indicates contraction and a vessel area > 100% indicates relaxation. To compare relaxation of pre-treated vessels, the vessel area was defined after pre-treatment again as 100%. Concentration-response curves of the vasodilators were performed and the effects reported as “Change [% of IVA]”. In the graphs, all pre-treatments were indicated. The luminal area of PAs/PVs was monitored with a digital video camera (Leica Viscam 1280, Leica DFC 280). The images were analysed with Optimas 6.5 (Media Cybernetics, Bothell, WA).

### Cyclic AMP and cGMP enzyme immunoassay

To analyse the role of cAMP/cGMP within imatinib-or nilotinib-induced relaxation, human PAs/PVs were isolated from tissue cores guided by their anatomical landmarks. PAs/PVs were incubated in medium, treated with imatinib or nilotinib (1 and 100 μM) and after ½ hour frozen in liquid nitrogen. Cyclic AMP/cGMP was quantified with ELISA-kits following the manufacturer’s protocol. For stabilisation, all samples or standards were acetylated. To measure cAMP levels, all samples were diluted 1:2 with 0.1 M HCL. The ELISA was evaluated at 405 nM (GENIOS, Tecan, Switzerland).

### Chemicals

Pentobarbital (Narcoren) was purchased from Merial (Hallbergmoos, Germany), gelatin from porcine skin from Sigma-Aldrich and low melting point agarose from GERBU (Heidelberg, Germany). ET-1 was purchased from BIOTRENDS (Wangen, Switzerland). Glibenclamide, iberiotoxin, 4-aminopyridine and amlodipine were purchased from Tocris Bioscience (Ellisville, Missouri, USA). Human PDGF-BB was provided by Peprotech (Hamburg, Germany). Imatinib and nilotinib were kindly provided by Novartis (Basel, Switzerland). Standard laboratory chemicals were obtained from Sigma-Aldrich (Steinheim, Germany). The ELISA-kits were acquired from Enzo (Lörrach, Germany).

#### Statistical analysis

**Fig. 5:**
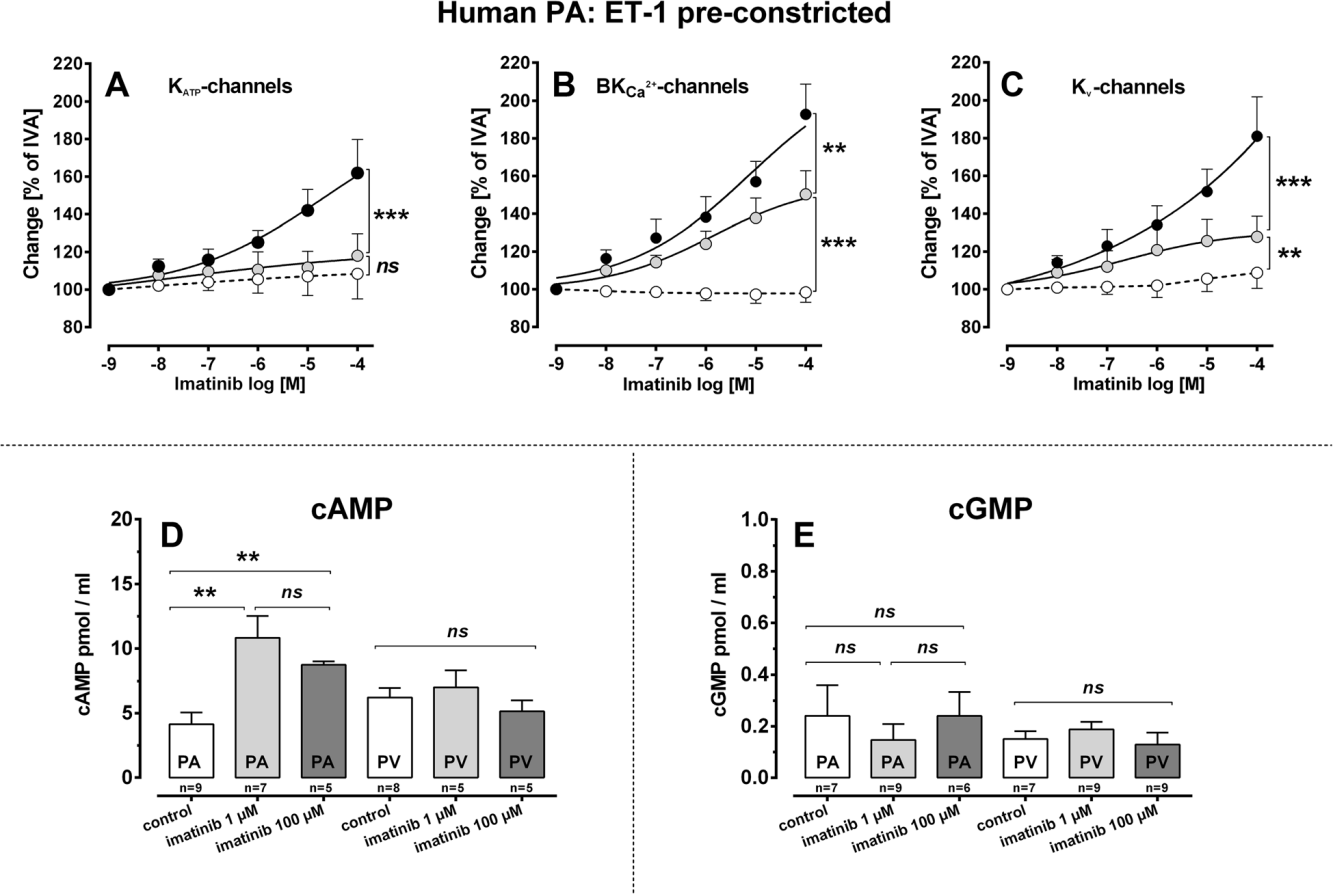
Involvement of K^+^-channels and cAMP/cGMP to the vasorelaxant effect of imatinib. **a** PA (100 nM ET-1): (●) imatinib (*n* = 5); () 10 μM glibenclamide / imatinib (*n* = 6); (◯) 10 μM glibenclamide (*n* = 7); **b** PA (100 nM ET-1): (●) imatinib (*n* = 5); () 100 nM iberiotoxin / imatinib (*n* = 4)(◯) 100 nM iberiotoxin (*n* = 5); **c** PA (100 nM ET-1): (●) imatinib (*n* = 6); () 5 mM 4-AP / imatinib (*n* = 6) (◯) 5 mM 4-AP (*n* = 6). **d** Effect of imatinib on cAMP. **e** Effect of imatinib on cGMP. **a-c** EC_50_ values were calculated using the standard 4-paramter logistic non-linear regression model. If EC_50_ values were not calculable (controls with K^+^-channel inhibitors), a LMM was applied. **d**, **e** Statistics were performed by the Mann-Whitney U test. *P* < 0.05 are considered as significant: ^*^ *p* < 0.05, ^**^ *p* < 0.01 and ^***^ *p* < 0.001

**Fig. 6:**
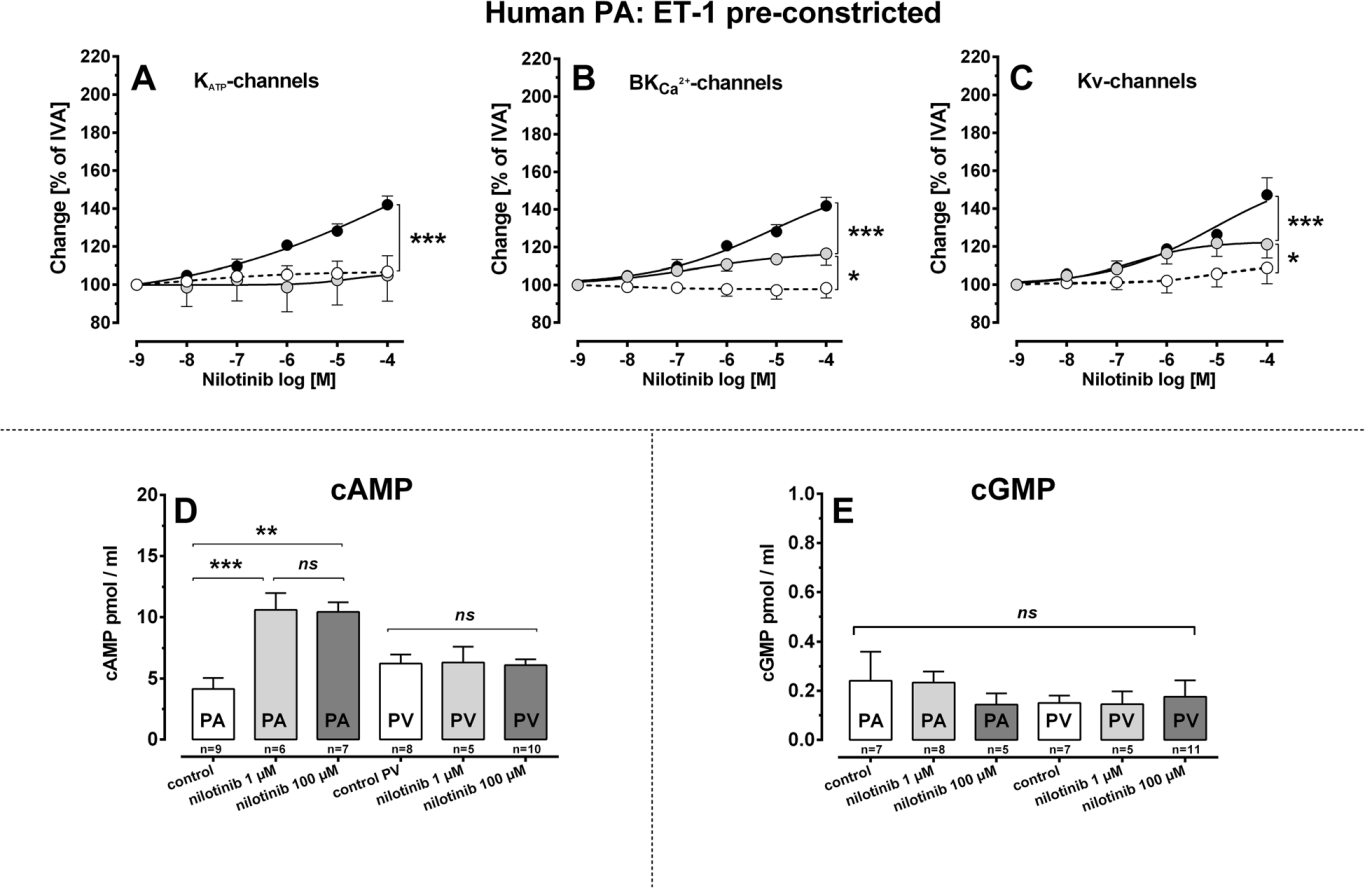
Involvement of K^+^-channels and cAMP/cGMP to the vasorelaxant effect of nilotinib. **a** PA (100 nM ET-1): (●) nilotinib (*n* = 6); () 10 μM glibenclamide / nilotinib (*n* = 4)(◯) 10 μM glibenclamide (*n* = 6); **b** PA (100 nM ET-1): (●) nilotinib (*n* = 6); () 100 nM iberiotoxin / nilotinib (*n* = 5) (◯) 100 nM iberiotoxin (*n* = 6); **c** PA (100 nM ET-1): (●) nilotinib (*n* = 6); () 5 mM 4-AP / nilotinib (*n* = 6)(◯) 5 mM 4-AP (*n* = 6). **d** Effect of nilotinib on cAMP. **e** Effect of nilotinib on cGMP. **a-c** EC_50_ values were calculated using the standard 4-paramter logistic non-linear regression model. If EC_50_ values were not calculable (controls with K^+^-channel inhibitors), a LMM was applied. **d**, **e** Statistics were performed by the Mann-Whitney U test. *P* < 0.05 are considered as significant: ^*^ *p* < 0.05, ^**^ *p* < 0.01 and ^***^ *p* < 0.001

**Fig. 7:**
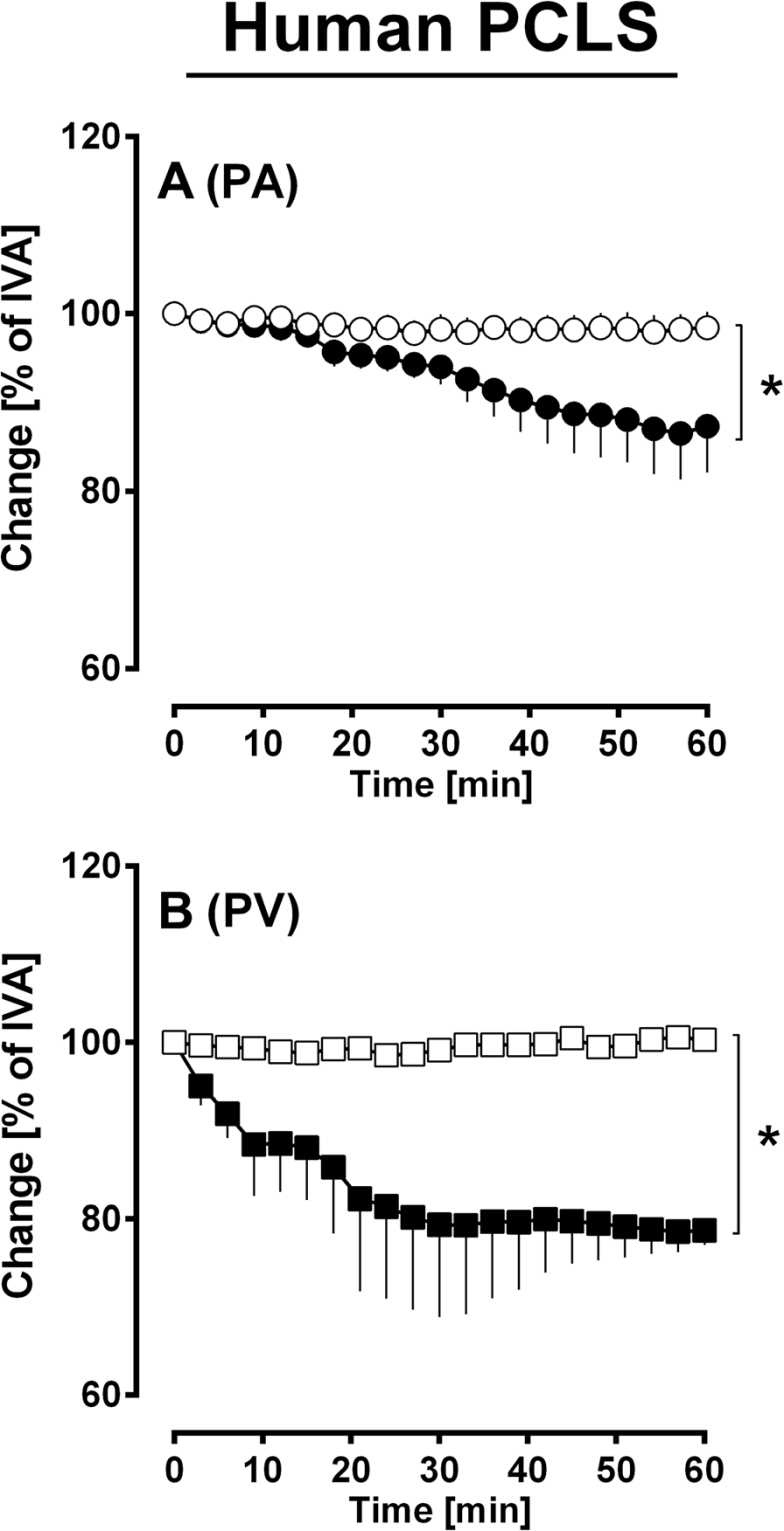
The contractile effect of PDGF-BB in human PAs and PVs. **a** Human PAs: (●) 100 nM PDGF-BB (*n* = 5); (◯) 100 μM imatinib / 100 nM PDGF-BB (*n* = 4). **b** Human PVs: (■) 100 nM PDGF-BB (*n* = 4); (□) 100 μM imatinib / 100 nM PDGF-BB (*n* = 3). **a**, **b** Statistics was performed by a LMM. *P* < 0.05 are considered as significant: ^*^ *p* < 0.05, ^**^ *p* < 0.01 and ^***^ *p* < 0.001

Statistical analysis was conducted using SAS software 9.3 (SAS Institute, Cary, North Carolina, USA) and GraphPad Prism 5.01 (GraphPad, La Jolla, USA). To analyse the data in Fig. 2b, d, Fig. 3b, d, Fig. 4b, Fig. 5b and Fig. 6a-c, EC_50_ values were calculated using the standard 4-parameter logistic non-linear regression model (GraphPad, La Jolla, USA). The AIC-criterion was used to select the most parsimonious model, i.e. a common bottom, top, slope and EC_50_ value in the regression model or the covariance matrix with the least number of parameters. Non-parametric analysis was performed by the Mann-Whitney U test; e.g. Figure 2f, g, Fig. 3e, f, Fig. 5d, e and Fig. 6d, e or by the Wilcoxon matched- pairs signed rank test (Fig. 2g) (GraphPad, La Jolla, USA). The data in Fig. 2c, h, Fig. 3c and Fig. 7a, b were analysed by a linear mixed model analysis (LMM) with the covariance structures VC or AR(1). The data in Fig. 5a-c and Fig. 6a-c were in part analysed by the LMM. *P*-values were adjusted for multiple comparisons by the false discovery rate and presented as mean ± SEM; (n) indicates the numbers of animals. *P* < 0.05 was considered as significant.

## Results

### Murine PCLS

In naïve PAs and PVs, imatinib did not exert relaxation (Fig. 2a). To enhance the vessel tone, PAs and PVs were exposed to increasing concentrations of ET-1 (100 pM to 10 μM) which contracted PAs or PVs up to 54% or 57% of IVA, respectively (Fig. 2b). For both PAs and PVs, the -logEC_50_ value was 6.8. PAs and PVs were exposed to 100 nM ET-1 which contracted them comparably (*p* > 0.05) over 90 min (Fig. 2c). After pre-constriction, imatinib relaxed PAs up to 167% of IVA with an EC_50_ value of 32 nM (Fig. 2d), whereas pre-constricted PVs did not relax to imatinib (Fig. 2e). Next, we exposed murine PCLS to 1 μM ET-1 which contracted PAs and PVs to 52% or 75% of IVA, respectively (data not shown). Afterwards, 10 μM imatinib slightly relaxed murine PVs up to 118% of IVA (Fig. 2f).

To study the effect of PDGF-BB in murine pulmonary vessel, they were treated with increasing concentrations of PDGF-BB (10^− 13^–10^− 7^ M). PDGF-BB at 100 nM contracted PAs up to 60% of IVA (Fig. 2g), whereas PVs only contracted to 86% of IVA (Fig. 2g). Next, PAs were exposed for 1 h to 100 nM PDGF-BB which contracted them up to 76% of IVA with a maximum 15 min after application (*p* < 0.001). Afterwards the contractile effect of PDGF-BB decreased, e.g. to 89% of IVA (*p* < 0.01) at 30 min (Fig. 2h). Finally, PDGF-BB treated PAs remained slightly contracted (Fig. 2h), although without statistical significance (*p* > 0.05). In order to study whether PDGF-BB-induced contraction is linked to PDGFR, we pre-treated PAs with 1 μM imatinib which prevented the contractile effect of PDGF-BB (Fig. 2h). Last we tried to find out, whether PDGF-BB-induced contraction depends on the activation of L-type Ca^2+^-channels and pre-treated PAs with 100 nM amlodipine which also prevented the contractile effect of PDGF-BB (Fig. 2h).

### Human PCLS – the effects of imatinib and nilotinib

#### Imatinib

Next we investigated whether imatinib could relax human pulmonary vessels. Naïve PAs and PVs did not relax to imatinib (Fig. 3a). To pre-constrict PAs/PVs, they were exposed to increasing concentrations of ET-1 (Fig. 3b). PAs and PVs contracted comparably to ET-1 (*p* > 0.05); with a -logEC_50_ of 7.5 for PAs and PVs (Fig. 3b). To prevent a different degree of pre-constriction, we pre-constricted PAs with 100 nM ET-1 and PVs with 50 nM ET-1 (Fig. 3c; *p* > 0.05). After pre-constriction, imatinib relaxed PAs with EC_50_ values of 3.8 μM, e.g. 1 or 100 μM imatinib relaxed PAs to 130% or 166% of IVA, respectively (Fig. 3d). For comparison, in oncological therapy, imatinib plasma levels of 1.8 μM are reached [34, 35]. Further, humans PVs relaxed to 107% of IVA which was statistical different from control PVs (*p* < 0.05) (Fig. 3e). In order to exclude that pre-constriction was too strong in PVs and thus imatinib-induced relaxation masked; we pre-constricted human PVs with 20 nM ET-1. Afterwards, 100 μM imatinib relaxed pre-constricted PVs to 116% of IVA (*p* < 0.05; Fig. 3f).

#### Nilotinib

In consequence of our results with imatinib, we studied whether the TKI nilotinib also relaxes human pulmonary vessels. Nilotinib had no effect in naïve human PAs or PVs (Fig. 4a). However, if human PAs were pre-constricted with 100 nM ET-1, they relaxed to increasing concentrations of nilotinib with EC_50_ values of 3 μM, e.g. PAs relaxed to 100 μM nilotinib to 145% of IVA and to 1 μM nilotinib to 119% of IVA (Fig. 4b). In humans, plasma levels of nilotinib of 0.7–5 μM are reached [36]. Unlike imatinib, nilotinib did not relax pre-constricted PVs (Fig. 4c).

### Human lungs - intracellular signalling

#### Imatinib: involvement of K^+^-channels

To study whether K^+^-channel-activation contributes to the relaxant effect of imatinib, we pre-treated human PAs with ET-1 and with one of the following K^+^-channel-inhibitors, e.g. 10 μg glibenclamide for K_ATP_-channels, 100 nM iberiotoxin for BK_Ca_^2+^-channels and 5 mM 4-AP for K_v_-channels (Fig. 5a-c). We did not perform these experiments in human PVs, as imatinib-induced relaxation was too weak. Simultaneous pre-treatment with ET-1 and glibenclamide, ET-1 and iberiotoxin or ET-1 and 5-AP did not alter the contractile effect of ET-1 in human PCLS (data not shown). However in human PAs, inhibition of K_ATP_-channels (glibenclamide) nearly prevented the relaxant effect of imatinib (Fig. 5a; *p* < 0.0001). Inhibition of BK_Ca_^2+^-channels (iberiotoxin) significantly reduced it (*p* < 0.01), as PAs relaxed to 150% of IVA in the presence of iberiotoxin compared to PAs with active BK_Ca_^2+^-channels which relaxed to 192% of IVA (Fig. 5b). In addition, inhibition of K_v_-channels (4-AP) also significantly reduced imatinib-induced relaxation (*p* < 0.01) and PAs only relaxed to 127% of IVA compared to 181% of IVA, if K_v_-channels were active (Fig. 5c). Thus, all three K^+^-channels mediate the relaxant effect of imatinib in human PAs, though K_ATP_-channels seem to be dominant.

#### Imatinib: involvement of cAMP/cGMP

Next we investigated whether imatinib could induce the generation of vasorelaxant second messengers such as cAMP or cGMP. Thus, we treated isolated human PAs and PVs with 1 or 100 μM imatinib (Fig. 5d, e). In human PAs, 1 or 100 μM imatinib increased cAMP (*p* < 0.01), though no difference was found between both concentrations (*p* > 0.05), whereas imatinib did not increase cAMP in human PVs (Fig. 5d). In addition, imatinib did not increase cGMP in human PAs or PVs (Fig. 5e).

#### Nilotinib: involvement of K^+^-channels

Accordingly to imatinib, we pre-treated human PAs with the K_ATP_-channel inhibitor glibenclamide (10 nM), with the BK_Ca_^2+^-channel-inhibitor iberiotoxin (100 nM) and with the K_v_-channel-inhibitor 4-AP (5 mM) in order to study the role of K^+^-channels within nilotinib-induced relaxation (Fig. 6a-c). Inhibition of K_ATP_-channels (Fig. 6a) prevented the relaxant effect of nilotinib (*p* < 0.001) and inhibition of BK_Ca_^2+^-channels (Fig. 6b) significantly reduced it (*p* < 0.001), as PAs only relaxed to 116% of IVA, an effect which differed (*p* < 0.05) from control PAs without nilotinib-treatment. Further, inhibition of K_v_-channels (Fig. 6c) reduced the relaxant effect of nilotinib (*p* < 0.001) and human PA only relaxed to 121% of IVA. This effect still differed (*p* < 0.05) from non-nilotinib-treated control PAs.

#### Nilotinib: involvement of cAMP/cGMP

As with imatinib, we analysed whether nilotinib increases intracellular cAMP and cGMP (Fig. 6d, e). In PAs, pre-treatment with nilotinib (1 or 100 μM) significantly increased cAMP (*p* < 0.01), but no effect was found in PVs (Fig. 6d). In addition, nilotinib did not increase cGMP in PAs/PVs (Fig. 6e).

### Human lungs - contractile effects of PDGF-BB in human PAs and PVs

The antiproliferative properties of imatinib rely on PDGFR-inhibition. Next we studied whether imatinib-induced relaxation also depends on PDGFR or conversely, if PDGF-BB contracts human PAs and PVs and if this contraction is prevented by imatinib. Therefore, we exposed human PAs and PVs to 100 nM PDGF-BB with or without imatinib-pre-treatment (Fig. 7a, b). PDGF-BB at 100 nM contracted human PAs (Fig. 7a; *p* = 0.01) and PVs (Fig. 7b; *p* = 0.04) and this contraction was prevented by imatinib (*p* < 0.05 for both).

## Discussion

TKI targeting the PDGFR kinases represent an intriguing approach to treat PAH, as they reverse the remodelling and improve pulmonary haemodynamics [5–7, 9–11, 16]. Beyond that, the antiproliferative aspects of TKIs appear to be promising in idiopathic pulmonary fibrosis [37, 38] or chronic asthma [39, 40]. The present study focused on the relaxant potential of TKIs, which was recently observed in PAs from rats [15, 16] and PVs from guinea pigs [17]. Here firstly, we report TKI-mediated relaxation of human and murine pulmonary vessel. Conversely, PDGF-BB contracted human and murine PAs/PVs. In human PAs, imatinib/nilotinib-related relaxation largely depended on K_ATP_-, BK_Ca_^2+^- and K_v_-channels. Last, imatinib/nilotinib increased cAMP which most probably contributes to their relaxant effect.

### The relaxant effect of imatinib and nilotinib

After pre-constriction, 10 μM imatinib relaxed murine PAs to 167% and human PAs to 148% of IVA. In human PAs, this effect was gradable to 166%, if 100 μM imatinib were applied. Finally, the relaxant effect of imatinib was most potent in murine PAs. In contrast, imatinib had only a weak relaxant effect in PVs of both species. Nilotinib (100 μM) relaxed pre-constricted human PAs to 145% of IVA; whereas pre-constricted PVs did not react. Our results suggest that 1) the relaxant effect of TKIs depends on the species, 2) the relaxant effect of TKIs varies within PAs or PVs and 3) there exist differences within the relaxant potential of imatinib or nilotinib.

#### Role of the species and the studied pulmonary vascular segments (PAs/PVs)

Here, the relaxant effect of imatinib varied across the species and along the pulmonary vascular bed. So, the impact of species, vessel size and anatomical affiliation is reconfirmed [31, 41–47]. We studied central murine PAs/PVs (diameter: 100–250 μm) and more peripheral human PAs/PVs (diameter: 500–1000 μm). This fact might account for the varying relaxant potential of imatinib in human and murine PAs/PVs, as in respect of the vessel size and the pulmonary vascular segment, PAs and PVs exert a certain K^+^-channel diversity [42]. The diversity of PAs and PVs is supported by their distinct behaviour to NO [41, 43], prostacyclin [44] or cardiotonic agents [31, 45, 46].

#### Role of pre-constriction within TKI-induced relaxation

We studied pulmonary vessels of a non-disease model. To pre-constrict PAs/PVs and to imitate the overexpression of ET-1-receptors and ET-1 in PAH [48, 49], we pre-constricted PAs/PVs with ET-1. In murine PAs/PVs, pre-constriction with 100 nM ET-1 contracted PAs and PVs comparably to 74 and 83% of IVA, respectively. In human PAs/PVs, 100 nM ET-1 contracted PAs to 44% of IVA and 50 nM ET-1 contracted PVs to 38% of IVA, which was also comparable. Although, pre-constriction was much weaker in murine pulmonary vessel, we did not use higher concentrations of ET-1, except from some experiments (Fig. 2f), as 1 μM ET-1 contracted murine PAs stronger than the corresponding PVs (data not shown). In general, ET-1-induced contraction strongly depends on the lot-number and on the age of the compound. Anyhow, murine PAs relaxed stronger than human PAs. The degree of pre-constriction certainly accounts for the potency of vasodilators. However, we cannot conclude that less or more pre-constriction is superior, e.g. human PVs pre-constricted with 20 nM ET-1 relaxed stronger to imatinib than PVs pre-constricted with 50 nM ET-1. In contrast, murine PVs pre-constricted with 100 nM relaxed less to imatinib than PVs pre-constricted with 1 μM ET-1. In this view, it is interesting that even comparable pre-constriction of murine (Fig. 2c) or human PAs/PVs (Fig. 3c), respectively, resulted in diverse responses. Beyond pre-constriction, other rationales such as K^+^-channel-density might account for the relaxant effects of imatinib in PAs/PVs [42, 50].

#### TKI-induced relaxation in dependence of their pharmacological profile

In human PAs, nilotinib relaxed with a weaker maximal effect than imatinib; e.g. 145% vs. 166% of IVA, respectively. Although, this difference is statistically not significant, it might be clinically relevant, as the consequential vascular resistance should vary. According to the Hagen-Poiseuille law, the flow resistance increases 16 fold, if the radius divides in half. Finally, the presented vascular effects of imatinib and nilotinib should be sufficient to be relevant for pulmonary vascular resistance.

The pharmacological properties of imatinib and nilotinib suggest that the relaxant potency of imatinib is superior. Firstly, both represent unselective PDGFR-antagonists inhibiting PDGFR-αβ with comparable IC_50_ values, e.g. 71 nM for nilotinib and 72 nM for imatinib [51]. However secondly, imatinib and nilotinib also inhibit the non-receptor tyrosine kinase c-Abl (ABL1) [52–54]. Within the several functions of c-Abl, the regulation of SMC-contraction by actin polymerisation is of high impact [55, 56], e.g. activation of c-Abl is involved within the contractile effect of PDGF-BB, as inhibition of c-Abl strongly attenuated PDGF-BB-induced contraction [20]. Both, imatinib and nilotinib inhibit c-Abl [51]. However, imatinib was approved to show a stronger docking score for the human c-Abl kinase receptor [57]. Due to this item, a stronger relaxant potency of imatinib seems to be conceivable.

### Role of K^+^-channel activation in TKI-induced relaxation

To highlight the mechanisms beyond imatinib/nilotinib-induced relaxation, we studied the role of K_ATP_-, BK_Ca_^2+^- and K_v_-channels. All of them are expressed in pulmonary vessels [42, 50]. Their stimulation hyperpolarises the cell membrane resulting in the reduction of cytosolic Ca^2+^-influx [58] and myosin light chain kinase (MLCK) activation [59]. Finally, vascular SMCs relax [59]. In human PAs, inhibition of all three K^+^-channels attenuated the relaxant effect of imatinib/nilotinib. Inhibition of K_ATP_-channels prevented it, whereas inhibition of K_v_-channels strongly reduced it. Further, inhibition of BK_Ca_^2+^-channels also decreased the relaxant effect of nilotinib/imatinib, anyway; human PAs still relaxed to a half-maximal response. The minor significance of BK_Ca_^2+^-channels within imatinib-induced relaxation goes in line with results in PVs from guinea pigs [17]; the superior role of K_ATP_-channels is supported by results from human prostatic SMCs [60, 61]. In contrast, results from Pankey et al. [16] did not reveal imatinib-related K^+^-channel-activation in the rat pulmonary arterial bed. Finally, this study shows that human PAs relax to imatinib/nilotinib due to the activation of K_ATP_-, BK_Ca_^2+^- and K_v_-channels.

### Role of cAMP and cGMP within TKI-induced relaxation

The tone of vascular SMCs is regulated by signalling cascades which finally modulate actin polymerisation and MLC-phosphorylation; e.g. by the increase of intracellular Ca^2+^-level or by Ca^2+^-sensitisation [62–64]. In this view, cAMP/cGMP plays a leading part. Cyclic AMP and its dependent protein kinase A (PKA) cause relaxation by K^+^-channel-stimulation. Further, cAMP acts in a Ca^2+^-desensitising manner: 1) cAMP blocks MLCK; 2) cAMP activates MLC-phosphatase (MLCP) [65]. Here, we showed that imatinib/nilotinib increases cAMP in human PAs; but not in PVs. This fact might explain in part the weak or missing relaxant effect of imatinib or nilotinib in human PVs, respectively. In a previous study in PVs [17], imatinib-induced relaxation also only depended on cAMP [17].

MLCP is activated by the cGMP-dependent protein kinase G (PKG) [65]. In addition, PKG also activates K^+^-channels [65]. Here, imatinib/nilotinib failed to increase cGMP in human PAs/PVs. This is in line with a previous study in PVs from guinea pigs [17], where imatinib 1) had no effects on cGMP-levels and 2) inhibition of NO-synthesis or PKG did not attenuate imatinib-induced relaxation. For nilotinib, no further data exist. Regarding imatinib, our results are supported by other studies [15, 16]. However, opposing data also exist, e.g. imatinib relaxed SMCs from human corpus cavernosum [60] and prostatic SMCs [61] in dependence to NO. In summary, the role of cGMP within imatinib-induced relaxation appears to depend on the tissue from which SMCs derive.

### The contractile effect of PDGF-BB in human and murine PAs/PVs

Corresponding to the relaxant effect of imatinib/nilotinib, PDGF-BB contracted pulmonary vessel from both species. Conversely, this effect was prevented by 100 μM imatinib. Finally, PDGFR regulates the tone of human and murine pulmonary vessels which is in line with a former study in PVs from guinea pigs [17]. In murine PAs, the contractile effect of PDGF-BB depends on calcium. The role of calcium for PDGF-BB-induced contraction is supported by studies in systemic vessels; e.g. rabbit isolated ear arteries [66] or rat aorta [67, 68] and pulmonary vessels; e.g. PVs from guinea pigs [20]. The contractile effect of PDGF-BB was comparable in human PAs and PVs (Fig. 7a, b; *p* > 0.05), although a trend towards a stronger effect in human PVs appears to be evident. This notice is somewhat unexpected, as imatinib-induced relaxation was weaker in PVs. In this view, unspecific mechanisms not related to PDGFR-activation are less probable, as imatinib prevented PDGF-BB-induced contraction. Other considerations include the activation of c-Abl, as it was shown for PVs [20]. In this regard, a varying activation of c-Abl in human PAs and PVs is conceivable, leading to a different degree of contraction.

## Conclusion

In conclusion, TKIs relax human and murine pulmonary vessels. Imatinib and nilotinib efficaciously relax human PAs by K_ATP_-, BK_Ca_^2+^- and K_v_-channel-activation. Here, 1 μM imatinib relaxed human PAs to 130% of IVA and 1 μM nilotinib relaxed human PAs to 119% of IVA. For comparison, during cancer therapy, imatinib [34, 35] and nilotinib [36] reach plasma levels of 1.8 μM and 0.7–5 μM, respectively. Hence, their relaxant effects appear to be of clinical relevance in the human pulmonary arterial bed and might be also of impact in PAH. With regard to imatinib-related side effects [10, 21], it is of clinical impact that nilotinib also relaxes human PAs and that TKIs also exert a relaxant effect via inhalation [17]. Anyhow, TKI-induced relaxation appears to be a class phenomenon which has been proven for several TKIs e.g. imatinib, nilotinib, sorafenib SU6668, DMPQ [15–17]. It includes with sorafenib also a TKI which exerts pulmonary vascular toxicity, whereas dasatinib was not shown to relax PAs and PVs. In view of PDGF-BB, we showed that PDGFR determines the tone of human and murine PA/PV and that this is preventable by imatinib. So, aside the antiproliferative effect of TKIs [5–14], this study confirms pulmonary vascular relaxation by PDGFR-antagonism. Recently, this dual action of PDGFR-antagonism was also shown in rats’ systemic vessels [16, 69]. In summary, TKIs are promising agents in PAH and other diseases with underlying proliferative and contractile pathophysiology.

## Data Availability

The datasets generated and analysed during the current study are available from the corresponding author on reasonable request.

## Abbreviation

4-AP: 4-aminopyridine
ET-1: Endothelin-1
IVA: Initial vessel area
LMM: Linear mixed model analysis
MLCK: Myosin light chain kinase
MLCP: MLC-phosphatase
PA: Pulmonary artery
PAH: Pulmonary arterial hypertension
PCLS: Precision-cut lung slice
PDGFR: Platelet-derived growth factor receptor
PKA: Protein kinase A
PKG: Protein kinase G
PV: Pulmonary vein
SMC: Smooth muscle cell
TKI: Tyrosine kinase inhibitor
